# A comparative study between cinnamon extract and cinnamon nanoparticles on growth performance, blood metabolites and gut microbiota of broiler chickens

**DOI:** 10.1016/j.psj.2025.106065

**Published:** 2025-11-05

**Authors:** Mokhtar Fathi, Parastoo Mardani, Abdoljabbar Shokri, Vahid Rezaee

**Affiliations:** a Department of Animal Science, Payame Noor University, Tehran, Iran; b Department of Biology**,** Payame Noor University, Tehran, Iran; c Department of Physic**,** Payame Noor University, Tehran**,** Iran

**Keywords:** Antioxidant, Broiler, Cinnamon, Nanoparticles, Microbiota

## Abstract

This study investigated the effects of dietary supplementation with bark powder extract of cinnamon and cinnamon nanoparticles on growth performance, antioxidant status, hematological parameters, and cecal microbiota in broiler chickens. A total of 500 one-day-old male Ross 308 chicks were randomly assigned to five treatment groups with five replicates of 20 birds each. The experimental treatments included a control (no additive), 200 and 400 mg/kg cinnamon extract (CIN-200 and CIN-400), and 200 and 400 mg/kg cinnamon nanoparticles (NANO-200 and NANO-400), administered via feed for 42 days. Results demonstrated that both forms of cinnamon significantly improved body weight gain and feed conversion ratio compared to the control (P < 0.05), with the highest gains observed in the NANO-400 group. Mortality rate was also significantly reduced in all supplemented groups. Antioxidant enzyme activities (GSH-Px, T-SOD, and CAT) were markedly increased, while MDA levels decreased, particularly in the NANO-400 group (P < 0.05). Hematological analysis revealed improved RBC count, hemoglobin concentration, and hematocrit levels, along with lower cortisol and HET/LYM ratios in NANO-400 group birds. Furthermore, the population of beneficial lactic acid bacteria increased significantly in the NANO groups, whereas *E. coli* counts were not significantly affected. In conclusion, dietary supplementation with cinnamon nanoparticles, especially at 400 mg/kg, effectively enhanced growth performance, antioxidant capacity, blood health, and beneficial gut microbiota in broiler chickens, suggesting its potential as a natural alternative to antibiotic growth promoters.

## Introduction

In recent years, increasing concerns over antibiotic resistance and consumer demand for chemical-free poultry products have stimulated the search for safe and effective alternatives to antibiotic growth promoters (AGPs) in broiler production. The use of medicinal plants as natural feed additives in broiler nutrition has gained increasing attention due to their potential to enhance growth performance, improve health status, and reduce reliance on antibiotics (Windisch et al., 2008; Hashemi & Davoodi, 2011). Medicinal plants and their bioactive compounds have received significant attention due to their antioxidant, antimicrobial, and growth-promoting properties (Hashemi & Davoodi, 2011).

Cinnamon (Cinnamomum verum), a widely used spice and medicinal herb, is rich in bioactive compounds such as cinnamaldehyde, eugenol, and polyphenols, which have been reported to enhance digestion, improve gut health, modulate lipid metabolism, and exhibit strong antibacterial and antioxidant effects (Ranasinghe et al., 2013; Al-Kassie, 2009; Salehi et al., 2019). Several studies have documented the beneficial impact of cinnamon powder or extract on broiler performance and immune responses (Hernandez et al., 2004; Akbarian et al., 2014; Rehman et al., 2018; Sarica et al., 2019).

However, the use of cinnamon powder or crude extract in poultry diets is often limited by poor bioavailability, low solubility, and instability under gastrointestinal conditions (Yin et al., 2019). These factors can reduce the effectiveness of cinnamon's active components and hinder its practical application. Extract forms of cinnamon generally offer better concentration and stability of bioactive compounds compared to the powder form, leading to improved physiological benefits in broilers (Abd El-Hack et al., 2020). Nanoparticles can improve cellular uptake and controlled release of active substances, thereby enhancing their efficacy (Prasad et al., 2017; Abdel-Daima et al., 2019; Kandeil et al., 2019; Sasidharan et al., 2020; Kumar et al., 2021; Sharifi-Rad et al., 2021). Cinnamon nanoparticles, produced via green synthesis or ultrasonic methods, have shown superior antimicrobial and antioxidant potential in vitro (Chaudhuri et al., 2014; Azizi et al., 2014;Yin et al., 2019), but they’re in vivo effects on poultry remain underexplored.

To date, few studies have compared the effects of conventional cinnamon and its nanoparticle form on the physiological and microbiological parameters of broiler chickens. This study addresses the research question: "Does dietary supplementation with cinnamon nanoparticles enhance growth performance and modulate gut microbiota more effectively than conventional cinnamon extract?" Unlike previous research focusing on cinnamon powder or crude extracts, this study evaluates nano-sized cinnamon particles, which may offer higher bioavailability and enhanced biological activity. We hypothesize that cinnamon nanoparticles will improve growth performance and positively influence gut microbiota. Therefore, the objective was to compare the effects of dietary supplementation with cinnamon and cinnamon nanoparticles on growth performance, blood metabolites, and gut microbiota composition in broiler chickens.

## Materials and methods

### Ethics statement

All animal-related experimental protocols were reviewed and approved by the Animal Ethics and Welfare Committee of the Department of Animal Science at Payame Noor University (Approval Code: IR.PNU.REC.1403.145). The entire study was conducted in strict compliance with the national ethical guidelines for the care and use of laboratory animals, as issued by the Iranian Ministry of Science, Research, and Technology. All necessary measures were undertaken to minimize animal suffering and to ensure the use of the minimum number of animals required to achieve statistical validity.

### Experimental birds and diet

A total of 500 one-day-old male broiler chicks (Ross 308) with an average body weight of 43 ± 2 g were randomly allocated to five dietary treatments in a completely randomized design. Each treatment consisted of five replicates with 20 birds per replicate (experimental unit). The five treatments were as follows: Control group (basal diet without additives), Basal diet + 200 mg/kg cinnamon powder, Basal diet + 400 mg/kg cinnamon powder, Basal diet + 200 mg/kg nano-cinnamon, and Basal diet + 400 mg/kg nano-cinnamon.

Birds were raised for 42 days in floor pens (2.2 m × 1 m × 1 m) bedded with wood shavings. For the first three days, bell drinkers were used, and then nipple drinkers were provided for the remainder of the experiment. Each pen was equipped with a separate 10-liter graduated, closed water container. The birds were fed starter (day 0–10), grower (day 11–24), and finisher (day 25–42) diets formulated based on corn-soybean meal to meet Ross 308 recommendations. The basal diets were formulated following the strain recommendations by Aviagen (2018), as specified in Table 1. Environmental conditions including temperature, humidity, and ventilation were maintained uniformly across treatments in accordance with the Ross 308 management guide. Vaccination against Newcastle disease was conducted via drinking water on days 7, 17, and 28, and against infectious bursal disease (IBD) on day 14.

**Table 1:** The ingredients and composition of the basal diet.

Ingredients (%)	Starter (0-10 d)	Grower (11-24 d)	Finisher (25-42 d)
Corn	52.86	57.58	61.76
Soybean meal, 48 %	39.1.0	33.98	30.10
Plant oil	3.72	4.41	4.41
Dicalcium phosphate.	1.82	1.63	1.40
Limestone	1.00	0.93	0.90
Salt	0.42	0.32	0.32
DL-Methionine	0.35	0.32	0.28
L-Lysin HCL	0.20	0.19	0.19
L-Threonine	0.13	0.11	0.11
Choline chloride 60 %	0.90	0.90	0.90
Sodium bicarbonate	0.10	0.14	0.14
Premix Blank	0.30	0.30	0.30
Total	100	100	100
Calculated values, %			
Metabolizable energy, kcal/kg	2977	3078	3130
Crude protein	23.37	21.23	19.66
Crude fat	6.04	6.78	7.00
Crude fiber	3.90	3.65	3.47
Calcium	0.93	0.85	0.77
Available phosphorus	0.49	0.44	0.40
Potassium	1.05	0.95	0.90
Sodium	0.18	0.14	0.14
Chloride	0.29	0.23	0.23
Dig. Methionine‏+cysteine	1.04	0.96	0.88
Dig. Lysine	1.35	1.21	1.12

Premix Per 1 Kg: 1400 IU Vitamin A, 3000 IU Vitamin D3, 50 mg Vitamin E, 4 mg Vitamin K, 3 mg Vitamin B6, 6 mg Vitamin B12, 60 mg Niacin, 20 mg Pantothenic acid, 0.20 mg folic acid, 150 mg Choline, 48 mg Ca, 3.18 mg P, 100 mg Mn, 50 mg Fe, 80 mg Zn, 10 mg Cu, 0.25 mg Co, 1.5 mg Iodine.

### Cinnamon and nano-cinnamon preparation

**Fig. 1:**
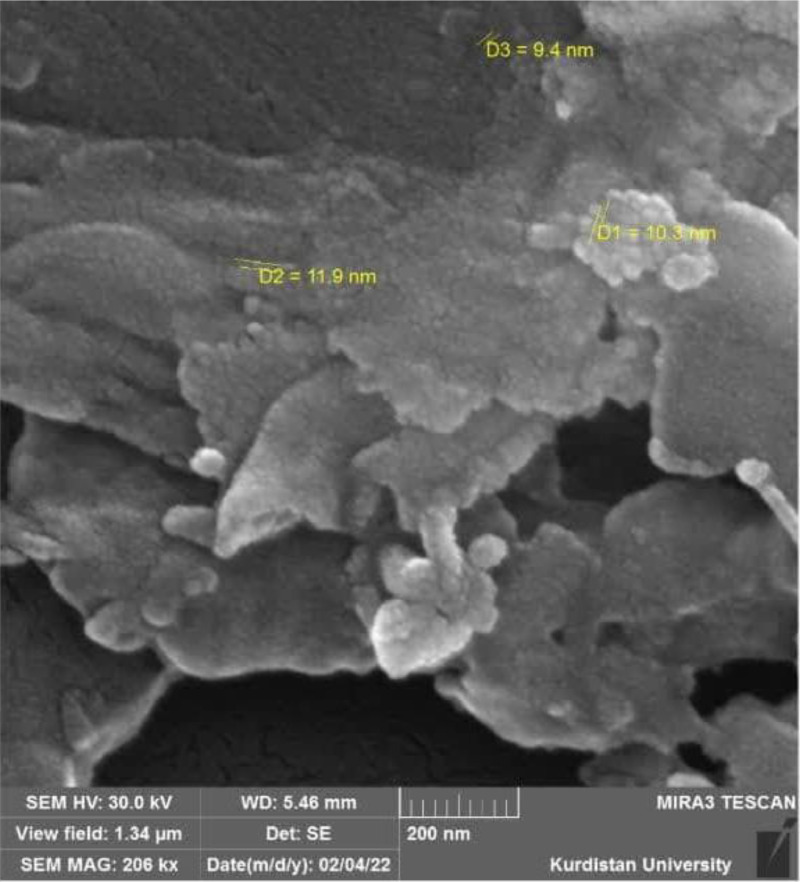
Electron microscope image of a cinnamon particle at different magnifications.

The cinnamon used in this study was a product of Adonis Gol Daru Company (Tehran, IRAN), packaged in a 1-kilogram bag of dark brown powder with a purity of 95 % and containing 10.31 % of the active ingredient cinnamaldehyde. The ethanol used was also obtained from Pars Medico Company with a purity of 99 %. Nano-cinnamon was synthesized using the ultrasonic method (cavitation technique) with a probe ultrasonic homogenizer (Bandelin SONOPLUS, 300 W, Faraz Teb Tajhiz, IRAN). In this method, 5 g of dry cinnamon extract powder (Adonis Gol Daru Co., 95 % purity, containing 10.31 % cinnamaldehyde) was dissolved in 400 mL of ethanol (PARS MEDICO, 99 % purity) and subjected to ultrasound waves at 25°C for 60 min. Among the different combinations tested, this condition resulted in the best dispersion and stability, while higher temperatures or concentrations led to agglomeration. The morphology and size of the synthesized particles were verified using scanning electron microscopy (SEM), which revealed spherical particles with sizes below 50 nm (Fig. 1). The proportion of nano-sized particles in the solution was determined to be 68 %.

### Broiler chicken's growth performance analysis

Body weights of individual broiler chickens were recorded on days 14 and 42 of the experiment. At the end of the trial, body weight gain (BWG) and feed intake (FI) were measured, and the feed conversion ratio (FCR) was calculated for the 28-day period. Daily health inspections were performed, and all mortalities were recorded along with their respective dates. FCR was determined by dividing total feed intake by body weight gain (FCR = FI/BWG), with adjustments made to account for the body weight of deceased birds, in accordance with the method described by Bahrampour et al. (2021).

### Blood Sampling for hematological and biochemistry indices

On day 42 of age, ten birds from each treatment group (two birds per replicate) were randomly selected and slaughtered following an overnight fasting period. Birds were euthanized via exsanguination, and blood samples were collected immediately. Serum was separated by centrifugation at 3,500 rpm for 12 min at 4 °C and stored at −20°C for subsequent analyses of biochemical and oxidative stress parameters. Hematological indices were measured using an automated hematology analyzer (Sysmex KX-21N, Japan). Differential leukocyte counts were performed manually according to the method described by Rasha et al. (2017). For further biochemical analysis, serum samples were again centrifuged at 2,500 × g for 10 min at room temperature and preserved at −20°C (Fathi et al., 2022). Serum concentrations of cortisol was determined using an automated biochemistry analyzer (Alcyon 300, Abbott, USA) with commercially available diagnostic kits (Pars Azmoon, Tehran, Iran).

Glutathione peroxidase (GSH-Px) activity was measured using the RANSel kit (RS-504, Randox Laboratories, Crumlin, UK). Catalase (CAT) and total superoxide dismutase (T-SOD) activities were determined using the RANSOD kit (SD-125, Randox Laboratories, UK). All enzyme assays were conducted according to the manufacturers’ protocols using the aforementioned autoanalyzer.

Malondialdehyde (MDA) concentration, an indicator of lipid peroxidation and oxidative stress, was quantified in serum following the method described by Fathi et al. (2022). MDA represents the end product of lipid peroxidation triggered by oxidative stress and was used as a biomarker to evaluate oxidative damage (Abdel-Daima et al., 2019).

### Intestinal microbiota

For microbial analysis, one bird per replicate (n = 5 per treatment) was euthanized by cervical dislocation on day 42. Ileal contents were aseptically collected and transferred to the microbiology lab. Serial dilutions (1:10 in sterile saline) were prepared, and samples were cultured on MRS agar for Lactobacillus *spp*. under anaerobic conditions and on MacConkey agar for Escherichia coli under aerobic conditions at 37°C for 48 h. Colony-forming units (CFU/g) were calculated by multiplying the number of colonies by the dilution factor (Asadi Siah Choghaei et al., 2022; Corduk et al., 2008).

### Statistical analysis

The collected data were analyzed using a one-way analysis of variance (ANOVA) for a completely randomized design, using SPSS software (version 19.0, SPSS Inc., IL, USA). Prior to analysis, the assumptions of normality and homogeneity of variance were tested using the Shapiro–Wilk test and Levene’s test, respectively. For growth performance parameters, the replication pen was considered as the experimental unit, while individual birds were treated as the experimental units for all other measurements. Differences among treatment means were compared using Duncan’s multiple-range test. Degrees of freedom (df), F-values, and exact P-values are reported for all analyses. A significance level of P ≤ 0.05 was used throughout the study.

## Results

### Growth performance and mortality

The effect of dietary supplementation of cinnamon extract and cinnamon nanoparticles on growth performance and mortality of broiler chickens at 42 days of age is shown in Table 2.

**Table 2:** Growth performance and mortality in broiler affected by dietary supplementation of cinnamon extract and cinnamon nanoparticles at 42 day of age.

Indices	Groups					SEM	P-Value
	Control	CIN-200	CIN-400	NANO-200	NANO-400		
Feed Intake [g]	3996.2^b^	4648.3^a^	4685.6^a^	4717.3^a^	47278^a^	81.66	0.020
Body weight gain [g]	2550.0^c^	2830.0^b^	2910.0^ab^	2930.0^a^	3070.0^a^	75.43	0.006
Feed conversion ratio [g/g]	1.83^a^	1.75^ab^	1.60^bc^	1.601^bc^	1.54^c^	0.027	0.033
Mortality rate (%)	4.00^a^	2.00^b^	0.00^c^	0.00^c^	0.00^c^	0.03	0.010

^a,b,c^ Mean values in the same row with different superscript letters were significantly. (n = 10).

CIN-200 and CIN-400, indicate the supplementation of cinnamon extract at the rate of 200 and 400 mg/kg respectively.NANO-200 and NANO -400, indicate the supplementation of cinnamon nanoparticles at the rate of 200 and 400 mg/kg respectively.

Feed intake was significantly affected by dietary treatments (P = 0.020). Birds fed diets supplemented with cinnamon nanoparticles and cinnamon extract had significantly higher feed intake compared to the control group, with the highest intake observed in the 400 mg/kg nanoparticle group (4727.8 g), while the control group had the lowest intake (3996.2 g). Broilers fed diets containing cinnamon nanoparticles at both levels (200 and 400 mg/kg) showed significantly higher (P = 0.006) body weight gain compared to the control group. The highest body weight gain was observed in birds receiving 400 mg/kg cinnamon nanoparticles (3070.0 g), followed by 200 mg/kg nanoparticles (2930.0 g), which were significantly higher than the gain in the control group (2550.0 g). Similarly, birds fed 400 mg/kg cinnamon extract (2910.0 g) had significantly greater weight gain than the control, while 200 mg/kg extract (2830.0 g) showed intermediate performance.

Feed conversion ratio (FCR) was significantly affected by the treatments (P = 0.033). The best FCR (lowest value) was observed in the NANO-400 group (1.54). Groups receiving nanoparticles at 400 and 200 mg/kg (1.54 and 1.60, respectively). FCR values in birds receiving cinnamon extract were intermediate (1.60 and 1.75 for 400 and 200 mg/kg, respectively). Mortality rate was significantly reduced (P = 0.010) by both cinnamon extract and nanoparticles. The highest mortality (4.00 %) was observed in the control group, while diets containing 200 and 400 mg/kg cinnamon nanoparticles and 400 mg/kg extract showed complete survival (0.00 %). Birds receiving 200 mg/kg extract had intermediate mortality (2.00 %).

### Antioxidant status

The effects of dietary supplementation with cinnamon extract and cinnamon nanoparticles on serum antioxidant status in broilers are presented in Table 3. Glutathione peroxidase (GSH-Px) activity was significantly higher (P = 0.010) in broilers supplemented with cinnamon nanoparticles at both 400 mg/kg (20.82 IU/L) and 200 mg/kg (20.56 IU/L) compared to those receiving cinnamon extract (19.21 and 18.10 IU/L for 400 and 200 mg/kg, respectively) and the control group (17.91 IU/L). Total superoxide dismutase (T-SOD) activity was also significantly elevated (P = 0.010) in the NANO-400 group (35.90 IU/L), outperforming all other treatments, with the control group showing the lowest activity (29.01 IU/L). Catalase (CAT) activity followed a similar pattern, with the highest activity observed in the NANO-400 group (13.39 IU/L), which was significantly greater than the control group (10.25 IU/L; P = 0.020).

**Table 3:** Antioxidant status in serum of broiler affected by dietary supplementation of cinnamon extract and cinnamon nanoparticles.

Indices	Groups					SEM	P-Value
	Control	CIN-200	CIN-400	NANO-200	NANO-400		
GSH-Px (IU/L)	17.91^c^	18.10^bc^	19.21^b^	20.56^a^	20.82^a^	0.85	0.010
T-SOD (IU/L)	29.01^c^	30.15^bc^	32.09^b^	31.16^b^	35.90^a^	1.02	0.010
CAT ((IU/L)	10.25^c^	12.49^b^	12.59^b^	12.16^ab^	13.39^a^	0.91	0.020
MDA (n mol/L)	2.98^a^	1.87^b^	1.65^b^	1.56^bc^	1.32^c^	0.08	0.012

^a,b,c^ Mean values in the same row with different superscript letters were significantly. (n = 10).

CIN-200 and CIN-400, indicate the supplementation of cinnamon extract at the rate of 200 and 400 mg/kg respectively.NANO-200 and NANO -400, indicate the supplementation of cinnamon nanoparticles at the rate of 200 and 400 mg/kg respectively.

GSH-Px, glutathione peroxidase; T-SOD, Total superoxide dismutase; CAT, catalase; MDA, malondialdehyde.

Malondialdehyde (MDA) concentration, an indicator of oxidative stress, was lowest in broilers fed NANO-400 (1.32 nmol/L) and highest in the control group (2.98 nmol/L), with significant differences observed (P = 0.012). Overall, supplementation with cinnamon nanoparticles, especially at 400 mg/kg, markedly enhanced the antioxidant defense system and reduced oxidative damage in broilers, demonstrating superior efficacy compared to cinnamon extract.

### Hematological parameters

The effects of dietary supplementation with cinnamon extract and cinnamon nanoparticles on hematological parameters of broilers are shown in Table 4. There were significant differences (P < 0.05) among treatments for RBC count, hemoglobin (HGB), hematocrit (HCT), heterophil percentage (HET), HET/LYM ratio, and cortisol levels. RBC counts were significantly lower in the NANO-400 (3.28) and NANO-200 (3.35) groups compared to CIN-400 (3.45), CIN-200 (3.47), and control (4.23) groups (P = 0.031).

**Table 4:** Hematological values of broiler affected by dietary supplementation of cinnamon extract and cinnamon nanoparticles.

Indices	Groups					SEM	P-Value
	Control	CIN-200	CIN-400	NANO-200	NANO-400		
RBCs count (× 10^6^/ μl)	4.23^a^	3.47^a^	3.45^a^	3.350^b^	3.28^b^	0.14	0.031
HGB (g/dl)	21.03^a^	19.51^b^	17.10^c^	18.1^bc^	17.92^bc^	0.65	0.021
HCT (%)	50.30^a^	43.01^b^	43.32^b^	42.20^b^	41.16^b^	1.24	0.040
WBCs count (× 10^3^/μl)	20.17	21.09	20.42	20.36	20.19	1.35	0.290
HET (%)	32.14	32.21^a^	32.01^a^	31.93^a^	28.72^b^	1.06	0.012
LYM (%)	66.13	64.29	65.17	65.20	66.09	1.43	0.420
HET / LYM ratio	0.48^a^	0.50^a^	0.49^a^	0.49^a^	0.43^b^	0.03	0.011
Cortisol (µg/dl)	19.25^a^	19.50^a^	19.01^a^	18.97^a^	15.32^b^	0.08	0.023

^a,b,c^ Mean values in the same row with different superscript letters were significantly. (n = 10).

CIN-200 and CIN-400, indicate the supplementation of cinnamon extract at the rate of 200 and 400 mg/kg respectively.NANO-200 and NANO -400, indicate the supplementation of cinnamon nanoparticles at the rate of 200 and 400 mg/kg respectively.

RBCs, Red blood cells; HGB, Hemoglobin; HCT, Hematocrit; WBCs, White blood cells; HET, Heterophil; LYM Lymphocyte.

Hemoglobin concentration (g/dl) was significantly lower in CIN-400 (17.10) than in CIN-200 (19.51), NANO-400 (17.92), NANO-200 (18.1), and control (21.03) groups (P = 0.021). Hematocrit (%) values were significantly reduced in all treatment groups (ranging from 41.16 to 43.32) compared to the control (50.30) (P = 0.040). There were no significant differences in WBC counts among the groups (P = 0.290).The percentage of heterophils was significantly lower in the NANO-400 group (28.72 %) compared to NANO-200 (31.93 %), CIN-400 (32.01 %), CIN-200 (32.21 %), and control (32.14 %) (P = 0.012). Lymphocyte percentages did not differ significantly among groups (P = 0.420). The HET/LYM ratio was significantly lower in the NANO-400 group (0.43) compared to other groups (0.48–0.50) (P = 0.011).

Cortisol levels (µg/dl) were significantly lower in the NANO-400 group (15.32) compared to NANO-200 (18.97), CIN-400 (19.01), CIN-200 (19.50), and control (19.25) groups (P = 0.023).

### Gut microbiota

The effects of dietary supplementation with cinnamon extract and cinnamon nanoparticles on the cecal bacterial population of broilers at 42 days of age are presented in Table 5. The populations of lactic acid bacteria were significantly influenced by the treatments (P = 0.021). Broilers supplemented with cinnamon nanoparticles at both 400 mg/kg (NANO-400) and 200 mg/kg (NANO-200) showed the highest counts of lactic acid bacteria (5.01 and 4.61 log10 CFU/g fresh digesta, respectively), which were significantly greater than those in the groups supplemented with cinnamon extract at 400 mg/kg (CIN-400), 200 mg/kg (CIN-200), and the control group (3.62, 3.60, and 3.15 log10 CFU/g, respectively). No significant differences were observed among treatments in the counts of *Escherichia coli* (P = 0.080), although numerical values tended to be slightly higher in nanoparticle-supplemented groups.

**Table 5:** Caecal bacterial population of broiler affected by dietary supplementation of cinnamon extract and cinnamon nanoparticles at day 42.

Microbiological Count (Log 10 CFU/g fresh digesta)	Groups					SEM	P-Value
	Control	CIN-200	CIN-400	NANO-200	NANO-400		
*Lactic acid bacteria*	3.15^b^	3.60^b^	3.62^b^	4.61^a^	5.01^a^	0.18	0.021
*E. coli*	6.95	7.01	7.12	7.21	7.18	0.43	0.080

*^a,b,^*^c^ Mean values in the same row with different superscript letters were significantly. (n = 10).

CIN-200 and CIN-400, indicate the supplementation of cinnamon extract at the rate of 200 and 400 mg/kg respectively.NANO-200 and NANO -400, indicate the supplementation of cinnamon nanoparticles at the rate of 200 and 400 mg/kg respectively.

CFU, Colony Forming Unit.

## Discussion

This study demonstrated that dietary supplementation with both cinnamon extract and its nanoparticle form positively influenced broiler performance, antioxidant capacity, hematological indices, and gut microbiota composition. Importantly, the nanoform of cinnamon showed consistently higher efficacy across most parameters, emphasizing the potential of nanotechnology in enhancing the functional properties of phytogenic feed additives.

The improvements in growth performance (BWG and FCR), particularly in the NANO-400 group, can be linked to enhanced nutrient utilization and intestinal health. This aligns with recent findings that nano-encapsulation increases the stability and bioavailability of phytochemicals, thereby improving digestion and absorption (Asadi et al., 2023).The superior efficacy of the nanoform likely arises from its increased surface area and solubility, which facilitate efficient cellular uptake and controlled release of active compounds (Anand et al., 2010). Such effects have also been reported in other plant-based nanoparticle studies in broilers, indicating improved feed efficiency and gut functionality (Ebrahimzadeh et al., 2020; Hussein et al., 2021).

Cinnamon’s key bioactives—cinnamaldehyde, eugenol, and cinnamic acid—are known to enhance digestive enzyme secretion, improve gut morphology, and exhibit strong antimicrobial and antioxidant properties (Kumar et al., 2013; Yang et al., 2015; Shen et al., 2022). These mechanisms collectively improve nutrient absorption and metabolic efficiency. The superior antioxidant response observed in NANO-supplemented birds, indicated by higher GSH-Px, T-SOD, and CAT activities and lower MDA concentrations, and further supports the role of cinnamon in mitigating oxidative stress. Nanoparticles may augment these effects by facilitating more efficient interaction with reactive oxygen species (ROS) and enhancing the expression of endogenous antioxidant genes (Kahya et al., 2020; Surai, 2016; Fathi et al., 2025).

Furthermore, the reduced H/L ratio and serum cortisol in NANO-fed birds indicate improved stress resilience. This suggests that cinnamon’s bioactive polyphenols, particularly in nanoform, contribute to immunomodulation and stress adaptation through antioxidative and anti-inflammatory pathways (Gross & Siegel, 1983; Yeh et al., 2013; Fathi et al., 2025). Improved redox balance likely supports better oxygen utilization and overall metabolic homeostasis, consistent with earlier reports on phytogenic antioxidants in poultry.

The modulation of gut microbiota, reflected in higher lactic acid bacteria counts and reduced pathogenic load, reinforces cinnamon’s antimicrobial selectivity. These effects are likely mediated by the bioactive compounds in cinnamon, especially cinnamaldehyde and eugenol, which disrupt bacterial membranes, inhibit pathogens, and selectively promote beneficial flora (Burt, 2004). In addition, cinnamon may modulate microbial metabolism and short-chain fatty acid production, further enhancing gut health and nutrient absorption (Zhang et al., 2022). The nanoform of cinnamon may provide greater stability and persistence in the gut environment, allowing improved solubility, bioavailability, and controlled release of active compounds, which collectively support microbial equilibrium (Hussein et al., 2021; Fathi et al., 2025). This balanced microbiota likely contributes to enhanced gut barrier integrity, reduced inflammation, and more efficient nutrient utilization, ultimately improving growth performance. Overall, the synergistic interplay between antioxidant, antimicrobial, and metabolic effects of cinnamon and its nanoform underpins the observed enhancements in broiler health and productivity. These findings highlight the potential of nano-encapsulated phytogenic compounds as effective and sustainable alternatives to antibiotic growth promoters in poultry production.

### Suggested limitations paragraph

Despite the promising results of this study, several limitations should be acknowledged. First, although cinnamon nanoparticles showed beneficial effects on growth, antioxidant status, and gut microbiota, their long-term safety for both birds and potential human consumers requires further investigation. Second, the environmental persistence of nanoparticles and their potential ecological impacts should be considered, as accumulation in soil or water systems may pose risks. Finally, the economic feasibility of large-scale application of cinnamon nanoparticles in commercial broiler production needs to be evaluated, including production costs and cost-benefit analysis compared to conventional feed additives. Addressing these limitations will be crucial for the practical and sustainable implementation of nanoparticle-based phytogenic feed strategies.

## Conclusion

Collectively, these findings confirm the potential of both cinnamon extract and especially cinnamon nanoparticles in enhancing broiler health and productivity. The superiority of the nanoform lies in its higher bioavailability, targeted delivery, and prolonged activity of phytochemicals. These results highlight the promise of phytogenic nanoparticles as sustainable feed additives in modern poultry production.

## CRediT authorship contribution statement

**Mokhtar Fathi:** Conceptualization, Data curation, Formal analysis, Project administration, Resources, Supervision, Validation, Writing – original draft. **Parastoo Mardani:** Investigation, Formal analysis. **Abdoljabbar Shokri:** Visualization, Validation, Software. **Vahid Rezaee:** Resources, Project administration, Methodology.

## Disclosures

We declare that we have no financial and personal relationships with other people or organizations that can inappropriately influence our work, and there is no professional or other personal interest of any nature or kind in any product, service and/or company that could be construed as influencing the content of this paper.
